# VAMP8 mucin exocytosis attenuates intestinal pathogenesis by *Entamoeba histolytica*

**DOI:** 10.15698/mic2017.12.605

**Published:** 2017-11-27

**Authors:** Steve Cornick, France Moreau, Herbert Y. Gaisano, Kris Chadee

**Affiliations:** 1 Department of Microbiology, Immunology and Infectious Diseases, Snyder Institute for Chronic Diseases, University of Calgary, Calgary, Alberta, Canada.; 2 Department of Medicine and Physiology, University of Toronto, Toronto, Ontario, Canada.

**Keywords:** Entamoeba histolytica, exocytosis, goblet cells, gut inflammation, innate immunity, mucin, pathogenesis

## Abstract

The intestinal mucosa encounters a barrage of ingested insults within the host yet under homeostasis elegantly facilitates nutrient absorption and sustenance of the commensal microbiota. An essential defence mechanism employed by the host is limiting the spatial niche various microbes may occupy as executed by the fluid mucus layer. Pathogens that violate their restricted niche within the intestinal mucosa are first expelled by robust mucus secretion from goblet cells thus by-passing the need for an immune response. Surprisingly, while many pathogens are known to exert hyper-secretion of mucus from goblet cells, the mechanisms governing this event remain elusive. In a recent report by Cornick *et al* (MBio 8: e01323-17), we nominate SNARE-mediated exocytosis as the putative mechanism responsible for pathogen-induced mucus secretion from goblet cells. The vesicle SNARE VAMP8 on mucin granules within goblet cells is specifically activated following infection with the protozoan parasite *Entamoeba histolytica* that is known to induce potent hyper-secretion and coordinates mucin exocytosis. This secretion event is critical in fending off a pathogen, as cells lacking VAMP8 are prone to increased *E. histolytica* colonization and cytolysis through apoptosis. Failing coordinated mucus exocytosis and subsequent epithelial barrier destruction, the host mounts an immune response as a last line of defence.

The colonic mucus barrier forms a bimodal layer consisting of an inner firmly adherent layer immediately above the epithelial cells and an outer less dense layer that is heavily colonized by commensal microbiota. Goblet cells continually replenish the mucus layer that is constantly eroded by luminal bacteria by producing and releasing the primary component of mucus, MUC2. Secretion of MUC2 from goblet cells has long been postulated to follow classical exocytosis despite no empirical evidence to suggest so. Indeed, the molecular machinery responsible for MUC2 release from goblet cells was not discovered until recently when Cornick *et al* described that exocytosis of mucin is heavily dependent on the SNARE vesicle protein VAMP8. Similar to neuronal exocytosis of neurotransmitters, SNARE mediated release of MUC2 utilizes the vesicle SNARE VAMP8 on mucin granules to likely bind cognitive SNARE receptors on the plasma membrane to facilitate membrane fusion. While constitutive release of mucin was dependent on VAMP8, pathogen-induced mucus hyper secretion was drastically reduced in VAMP8 deficiency.

The protozoan parasite *E. histolytica* responsible for amebiasis is a colonic pathogen that presents asymptomatic in most individuals. We hypothesize that *E. histolytica* remains restricted to the luminal niche in these individuals and once the parasite breaches the mucus layer to interact with epithelial cells it exerts a pathogenesis profile to invoke disease in a small subset of infected hosts. The integrity of the mucus layer therefore serves as a critical first line of innate host defence against invasion by *E. histolytica* and indeed mice lacking Muc2 mucin display a more severe disease. Similarly, VAMP8-mediated mucin exocytosis is equally important during infection with *E. histolytica,* as mice lacking Vamp8 have increased apoptosis and cytolysis of epithelial cells due to aberrant mucin release. This followed classical apoptosis pathways as characterized by Caspase 3, 9 and PARP cleavage. Clearly this demonstrates the importance of both constitutive mucin release to maintain the barrier as well as induced mucin secretion in response to a pathogen to fend off imminent threats. *E. histolytica* therefore serves as an important model to investigate the role of coordinated VAMP8-dependent SNARE exocytosis in goblet cells that has broad implications to many intestinal pathogens that induce mucus secretion. It is likely to conceive that genetic susceptibility loci that alter the ability of goblet cells to execute proper mucin exocytosis would therefore render the host susceptible to infection from a variety of intestinal pathogens.

Perturbation of the mucin secretion SNARE machinery has detrimental effects on the ability of the host to innately protect against luminal pathogens; a burden that is ultimately passed to members of the myeloid compartment to control an invading threat. Indeed, in mice lacking Vamp8 we noted a more aggressive pro-inflammatory cytokine release characterized by Il-1α, Il-1β and Tnf-α compared to mice with intact mucin exocytosis. In the context of *E. histolytica*, invading parasites are well poised to subvert inflammatory macrophages and translocate to extra-intestinal sites such as the liver and brain. A synopsis of how VAMP8 deficiency leads to abrogated mucin release from goblet cells and the downstream consequences is summarized in Figure 1.

**Figure 1:**
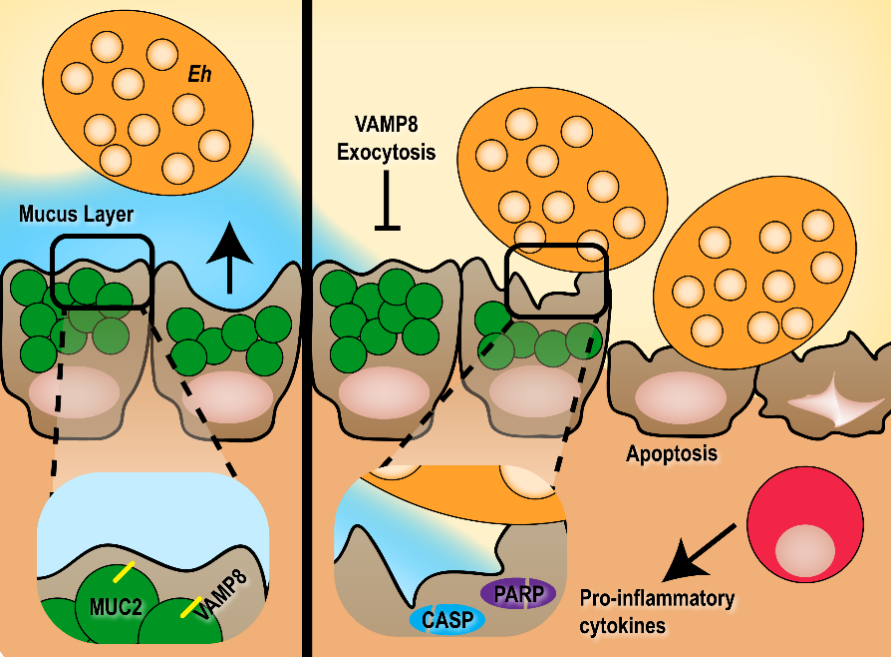
FIGURE 1: The pathogenesis of *E. histolytica* in the absence of VAMP8-mucin exocytosis. Under normal conditions (left), *E. histolytica* (*Eh*) colonizes the mucus layer and induce VAMP8-dependent mucin exocytosis when in close proximity to goblet cells. This hyper secretion of mucin is essential in restricting pathogen contact with the epithelium in a normal host-pathogen relationship. However, if mucin exocytosis is blocked through VAMP8 deficiency (right), the host cannot adequately fend off the parasite leading to direct contact with mucosal epithelial cells and induction of apoptosis through caspase 3, 9 and PARP cleavage evoking an acute pro-inflammatory response in disease pathogenesis.

The study reviewed here highlights the first report of regulated SNARE exocytosis in goblet cells to afford mucin secretion. While the study focuses on disease pathogenesis, the consequences of VAMP8 deficiency on abrogating mucin exocytosis constitutively to maintain homeostasis remains elusive. Further studies should evaluate the plasma membrane SNARE receptor complex implicating an involvement for SNAP and Syntaxin core machinery proteins. Additionally, while the role of kinases in driving mucin secretion in goblet cells is established following *E. histolytica* infection we do not know how these kinases activate the SNARE complexes. Analogous to other models of non-neuronal exocytosis, this event likely stems from phosphorylation of SNARE proteins and/or chaperones.

